# Is transactional sex exploitative? A social norms perspective, with implications for interventions with adolescent girls and young women in Tanzania

**DOI:** 10.1371/journal.pone.0214366

**Published:** 2019-04-02

**Authors:** Joyce Wamoyi, Lori Heise, Rebecca Meiksin, Nambusi Kyegombe, Daniel Nyato, Ana Maria Buller

**Affiliations:** 1 Department of Sexual and Reproductive Health, National Institute for Medical Research, Mwanza, Tanzania; 2 Department of Population, Family and Reproductive Health, Johns Hopkins Bloomberg School of Public Health, Baltimore, MD, United States of America; 3 Department of Public Health, Environment and Society, London School of Hygiene and Tropical Medicine, London, United Kingdom; 4 Department of Global Health and Development, London School of Hygiene and Tropical Medicine, London, United Kingdom; University of California San Diego, UNITED STATES

## Abstract

Although transactional sex is common in many sexual relationships, there has been little research into the degree to which the practice is considered exploitative in the settings in which it is practiced. We describe the social norms that influence transactional sex in two sites in Mwanza, Tanzania, and explore local understandings of whether and under what conditions it is considered exploitative. We then compare these “emic” understandings of exploitation to international definitions and norms around sexual exploitation. This study employed a qualitative research design involving 18 focus group discussions and 43 in-depth interviews with young people aged 14–24 years and parents with children aged 14–24 years in a rural area and an urban center within Mwanza, Tanzania. Thematic analysis was conducted with the aid of NVivo 10. The social norms influencing the practice of transactional sex included: reciprocity as a core cultural value that permeates the way exchange in sexual relationships is judged; gendered expectations that men should provide for women’s material needs in sexual relationships and that women should reciprocate by means of sex; and peer pressure to be perceived as “fashionable”. Adolescent girls and young women (AGYW) are under strong peer pressure to conform to a “modern lifestyle” as reflected in stylish clothing and other items of modernity such as cellphones. The emic conceptualization of exploitation is defined by circumstances surrounding the relationship or a sexual encounter. Important factors that characterize local notions of when transactional relationships are considered exploitative include: when the encounter or relationship involves an imbalance of power (based on age, male economic power and social status); when a man fails to reciprocate; and when sex is coerced. According to community perspectives, young women’s behavior should be considered exploitative of men when they take gifts or money yet refuse sex or when they demand large sums of money. Interventions aimed at reducing AGYW’s exploitation through transactional sex need to be cognizant of the variations in the understanding of what constitutes sexual exploitation as well as the social and gender norms influencing the practice of transactional sex. Interventions need to involve communities and families in critical thinking that helps them identify positive alternatives to current gendered social norms that shape the involvement of AGYW and men in transactional sex.

## Introduction

Evidence from sub-Saharan Africa shows that transactional sex is prevalent in the relationships of adolescent girls and young women (AGYW) [1–5]. Transactional sex has been defined in myriad ways but, for the purposes of this article, we define it as non-marital, non-commercial sexual relationships, motivated by the implicit assumption that sex will be exchanged for material support or other benefits [4]. The meanings attached to the exchange vary from one individual to another, with key motivations being to meet basic needs and to acquire items of modernity [3, 6, 7]. Gender inequalities shape sexual relationships by giving men greater social and economic power than women and create the material and ideological conditions that encourage transactional sex [6–8]. Evidence shows that, because of widespread societal expectations that men should provide and women should receive and reciprocate with sex, it is the lack of transaction in sexual relationships that is perceived negatively [5, 9]. Hence, AGYW enter into sexual relationships expecting to receive something from their partner [5, 8, 10].

From a public health perspective, transactional sex has been found to increase the risk of HIV and other sexually transmitted infections, and can also result in unplanned pregnancies as well as other undesirable sexual and reproductive health outcomes, such as intimate partner violence, sexual coercion and increased alcohol consumption among AGYW [11–16]. When transactional sex involves young girls and older partners, the consequences can be more detrimental, as power to negotiate use of family planning, condom use and *whether* and *when* to have sexual intercourse is further hampered [7, 17, 18]. Hitherto, most efforts undertaken to address transactional sex have emerged from the field of HIV prevention, usually prioritizing knowledge provision and change of individual attitudes as primary drivers of change. These interventions have paid less attention to the psychosocial and economic drivers and consequences of transactional sex for young girls, such as decreased access to resources and education, which reinforces the cycle of poverty in which girls who engage in transactional sex are usually locked. Likewise, structural, cultural and community-level factors that may underpin such gender norms and power imbalances in relationships are usually under-theorized.

From a human rights and child protection perspective, sexual exploitation of children and adolescents involves situations, contexts and relationships whereby individuals under the age of 18 (or a third person or persons) receive “something” (e.g. food, accommodation, drugs, alcohol, cigarettes, affection, gifts, money) in return for performing sexual activities and/or having others perform sexual activities on them [19]. In this context, exploitation is understood to mean the actual or attempted abuse of a position of vulnerability, differential power or trust, for sexual purposes [20]. International frameworks derived from this perspective, such as the United Nations Convention on the Rights of the Child, consider these exchanges to be a form of child sexual exploitation when the encounter involves an adult and a young person below the age of 18 [20, 21]. However, according to a well-established body of literature arising from the African context [5, 17], AGYW involved in these relationships and others within their communities may not perceive these relationships to be exploitative, demonstrating that these definitions are not always as clear-cut in practice.

Bearing the above in mind, this article aims first to explore local social norms influencing transactional sex in the context of Tanzania and second to identify the community (emic) perspectives on if and when transactional sex relationships are considered exploitative. By pursuing these objectives, it adds to the body of literature providing evidence-based insights to inform the design of interventions to prevent exploitative transactional sexual relationships and/or reduce risks inherent to the practice.

### Theoretical framework

Using a social and gender norms theory framework, we set out to explore the norms influencing transactional relationships and how existing gender and social norms prevailing in the study setting directly modify local understandings of exploitation. Social norms are defined as informal rules that define appropriate behavior in a given context, and vary across time, place and population [22]. People comply with norms for various reasons [22], including the desire to be accepted within their community, family and peer groups (referred to as “reference groups” in social norms theory) and the fear of social disapproval and rejection if they do not comply with the norm. For our purposes, gender norms are the widely accepted social rules about roles, traits, behaviors, status and power associated with being a man or considered masculine or being a woman or considered feminine in a given culture [23].

We envisage that individuals’ respective beliefs about the prevalence and meaning of transactional sex and sexual exploitation are instrumental to their engagement in and experience of these behaviors. We hypothesize that AGYW’s decision to engage in transactional sex, their views on what sexual exploitation is and their perspectives on whether or not to take action when it occurs are very much determined by the social norms around the practice, in particular gender norms governing relationships and interactions between men and women.

We conceptualize that AGYW’s engagement in transactional sex is in part shaped by the beliefs they hold about how prevalent transactional sex is in their community, if they think others in their community expect them to engage in transactional sex and, very importantly, the perceived social rewards of engaging in it. Following this line of thought, AGYW and other community members’ perspectives about “exploitation” and how they conceptualize it are also likely to be influenced by their beliefs about what others in their reference group think constitutes exploitation and what ought to be done in the case of exploitation in their community.

## Methods

This study employed an observational qualitative design, involving focus group discussions (FGDs) (combined with vignettes) and in-depth interviews (IDIs). Fieldwork was undertaken in two sites in the Mwanza region (one rural and one urban) of Tanzania. The study population included male and female adolescents and young people, aged 14–24 years, and adult community members, aged 25–49 years, within the study sites who had children within the age range of 14–24 years.

### Sampling and data collection

The sites and participants were sampled to reflect a broad range of experiences and different demographic profiles using snowball and purposive techniques. After a meeting with the ward and village authorities, the researchers were introduced to three unmarried young women and three unmarried young men who were out of school; and three young women and three young men who were in school. Initial contacts were asked to introduce the researchers to their network of friends, who were in either the in-school or the out-of-school category.

As Table 1 indicates, a total of 18 FGDs (with an average of 10 people per group) were conducted. A total of 43 IDIs (15 with young women, 8 with young men, 12 with adult men, 8 with adult women) sampled from within the FGD participants were also conducted. Adult men and women with children aged 14–24 years were selected to participate in the study because we wanted to collect their views on daughters’ potential engagement in transactional sex and whether or not they thought girls in these types of relationships were being exploited or not.

**Table 1:** Overview of focus group discussions and in-depth interviews.

	FGDs (n = 18)		IDIs* (n = 43)	
Participant category	Rural	Urban	Rural	Urban
Young women aged 14–24*				
In school	1	1	4	3
Out of school	2	2	4	4
Young men aged 14–24*				
In school	1	1	2	2
Out of school	1	1	2	2
Adult women aged 25–49*	2	2	6	6
Adult men aged 25–49*	2	2	4	4
**Total**	**9**	**9**	**22**	**21**

*All parents and adolescents who participated in an IDI had previously participated in an FGD

FGDs were instrumental in exploring discourses around sexual exploitation and the meanings attached to such behaviors. Questions were limited to general views and no personal experiences were discussed. The FGDs explored: motivations for and consequences of transactional sex; norms and expectations of transactional sex; readiness for sex; girls as targets of male sexual interest; understanding of sexual consent; and sexual exploitation. At the end of each FGD, vignettes were used to elicit participants’ views on whether they thought sexual encounters in different scenarios were “right” versus “wrong”; “fair” versus “unfair”; and “good” versus “not good”.

The IDIs played a complementary role to the FGDs by contributing to the understanding of AGYW’s transactional sexual relationships and the exploitative aspects of these. IDI participants were selected from within the FGDs based on their willingness to participate and their enthusiastic participation or lack of it, as reflected during the FGD sessions. By reflecting on the responses from the FGDs, the IDIs focused specifically on gaining a rich understanding of personal views. Similar to the FGDs, the IDIs followed a semi-structured topic guide, making it possible for the researcher to explore questions not included in the guide but of relevance to the study as they emerged. This open-ended, discursive approach allowed for an iterative process of refinement, whereby lines of thought identified by earlier interviewees were taken up and presented to later interviewees [24].

Both the IDIs and the FGDs were conducted in a private place selected by participants in collaboration with the researcher. Given the sensitivity of this topic and the cultural tradition in the study context, participants were matched to interviewers by sex. IDIs took approximately one and a half hours; FGDs took a maximum of two hours. IDIs and FGDs were audio-recorded. The interviewers for this study were trained social scientists with Bachelors or Masters degrees and who had experience collecting sexual behavior data. Ethical clearance for the study was obtained from the Medical Research Coordination Committee of the National Institute for Medical Research and the London School of Hygiene and Tropical Medicine. Oral and written informed consent was obtained from all participants. For children below the age of 18 years, parental consent and child assent were obtained.

### Data analysis

Data were transcribed verbatim, translated into English, entered into QSR NVIVO 10 software and coded by two researchers involved in the data collection. A pragmatic approach to analysis was adopted, involving combined use of an already designed coding scheme (anticipated codes) and grounded codes. Grounded codes were developed by means of a thorough reading of the data by two researchers in consultation with the data collection team and reflected the language and ways of expressing ideas portrayed by the participants. The anticipated codes were developed from the research objectives, prior knowledge and repeated reading-through of the data during the early stages of the analysis, and then refined in light of further data. Code refinement was conducted in consultation with the research investigators during a data analysis workshop.

The first author then thoroughly examined the coded data for emerging patterns. For example, the connection between concepts such as transactional sex and “exploitation” was examined by exploring codes on how participants talked about transactional sex and “exploitation” in FGDs and in IDIs. Theories were formulated, such as: “the way adults and young people talked about exploitation depends on the circumstances of the sexual encounter and the perceived behavior of the girl involved.” In order to test this theory, codes relating to exploitation and transactional sex were searched, summarized and compared. Widespread views supporting the emerging theories were examined alongside the deviant cases. In the presentation of results, deviant cases are also presented as appropriate. Quotations illustrating the main findings were identified.

## Results

### Description of AGYW’s sexual relationships

Sexual debut among girls happens between the ages of 9 and 15 years; among boys it occurs between the ages of 14 and 17 years. The majority of adolescent girls reported that they were sexually active and thought their peers were as well.

AGYW’s sexual partners range from peers to much older men (5–10 years older), and each category of partner plays different roles. The motivation for having peer partners is emotional as well as academic support at school, whereas older partners are mainly for material support. Items male sexual partners commonly give to AGYW are: money, clothes, shoes, cellphones, school requirements, books, motorbike transport and household items, including food. Exchange also entails non-tangible elements such as grades in exchange for sex in the case of students and teachers. Although a few AGYW reported engaging in a sexual relationship for emotional reasons such as love, these were always expressed through the provision of tangible and non-tangible items received from their sexual partner.

### Gender norm of male provision

The social expectation that men should provide for women’s material needs in sexual relationships is widespread. Participants described the norm of male provision as having been in existence for many generations. However, they noted some variations in the nature of exchanges within contemporary and traditional relationships.

*This issue of men giving to women things has been there since time immemorial*. *The difference between the past and present is how the exchange is given*. *Now we give each other money and things from the shops but in the past they gave sugarcane*, *maize*, *goats [laughter]*. *[FGD*, *out-of-school young men*, *urban]*

Provision is linked to masculinity, with men using phrases like “a true/total man” to refer to those who provide for their partner. Moreover, ability to provide is considered a sign of future responsibility and hence makes a man attractive as a potential marriage partner. A young man described this in the following:

*Even before entering in a relationship*. *A “real” man is seen even before starting a family*. *If you cannot provide for your girlfriend it is clear that even after marrying her*, *your family will be sleeping hungry [inability to provide]… Because you cannot work hard to get money [IDI*, *out-of-school young man*, *20 years*, *urban]*

When girls were asked for their views on whether they could stop engaging in transactional sex if all their material needs were provided for, the majority reported that they would still expect to receive something from their male partner. They mentioned that it was men’s responsibility to provide for women regardless of the latter’s economic status.

*He must give me… Yes*, *it is obvious that the men must give women their needs… So the man would just give a woman gifts*. *[IDI*, *out-of-school young woman*, *20 years*, *urban]*

Since provision was always explained as men’s responsibility, most men reported that they needed to have money before starting a sexual relationship. A young man talked about his provider role as including material and emotional elements:

*To be honest*, *I can say my biggest responsibility is to provide for my partner as much as I can*, *for example giving her gifts*, *encouraging her when she is stressed and advising her on life*. *It is just things like those*. *[IDI*, *out-of-school young man*, *19 years*, *urban]*

Likewise, girls are under pressure to expect and obtain gifts and money from their male partner. Girls talked about peers ridiculing peers who had not received anything from their sexual partner and referring to them using stigmatizing labels such as “backward”, “not loved” and “useless”. Girls equated male provision within their sexual relationship to an expression of love and hence perceived peers who did not receive gifts and money from their partner as not loved. Participants in a discussion with out-of-school girls described the ridicule of girls who had not received gifts:

*Her peers laugh at her because she is not given things*. *They think that she is very naïve or backward/not trendy (mshamba sana)… because you have a boyfriend*, *but he doesn’t give you things*, *not even money… “You feel like he doesn’t love you*, *maybe there is someone whom he loves and gives his money”… They just gossip about you*. *You may feel as if you are not worth among your friends*. *Maybe you see them with a new pair of sandals and ask them*, *they would tell you “Why don’t you also buy*? *She discloses “So and so doesn’t give me money”* … *They will advise her that “You should be asking him” [FGD*, *out-of-school girls*, *rural]*

### Reciprocity, complacent femininities and sexual relationships

Another widespread cultural norm that seems to permeate the way sexual relationships are judged is that of reciprocity—the idea that if you receive something from someone you should give something in exchange. Hence, almost all participants reported that it was expected for girls to receive something from their male partner in exchange for sex. So, while masculinity is linked to male provision, with “good men” expected to provide their partner with gifts and money, “good women” are expected to reciprocate with sex, and, as the following quote shows, failure to reciprocate is frowned upon:

*First of all*, *I consider a man who does not provide cruel… Even if the girl is very desperate*, *she would rather just stay without one than to have sex for nothing… you can’t get involved in sexual relationship with a man who doesn’t give you anything*. *But if the man gives her and she is capable of investing in a business that will help her improve her life*. *But if he does not help her it is not good*. *[IDI*, *out-of-school young woman*, *18 years*, *rural]*

A further attestation to the norm of reciprocity is the belief that girls who attract men who do not offer anything in exchange are unlucky. Such girls face stigma from their peers. They are looked down upon and do not attract as many friends as those who have nice things and are perceived as lucky.

*For that one who gets nothing*, *they say she has misfortunes*. *There are some*, *you find that a man spends a night with her and doesn’t give her anything*. *He just plays with her and leaves her… She is just unlucky*. *[FGD*, *adult women*, *urban]*

Although reciprocity is widely accepted, in a few instances young women questioned the expectation for girls to give sex in return for the gifts and money they received from men. They considered this an unfair exchange. A school girl with a contrary view said:

*It is not right… it is not that when he gives he has to get something in return*… *when he offers to help you he should do it whole-heartedly*. *[IDI*, *in-school young woman*, *17 years*, *urban]*

### Globalization norms and peer pressure for a trendy life style

Entering into transactional sex provides AGYW with social acceptance among peers. The majority of AGYW are under strong peer pressure to conform to “modern” lifestyles, commonly referred to as “going with the times” (*kwenda na wakati*). AGYW’s desire for trendy clothing and items of modernity often pushes them into relationships with older partners so they can afford these items. Young people and adults mentioned items such as: cellphones, tight knee-length trousers known as “pedal pushers” and plaiting hair.

*The things that contribute a lot [to engaging in transactional sex] are the desire to have these pedal pushers*, *tights and braiding hair in saloons… girls of these days also desire to have phones so that they can chat with someone… they want to be like others around them [FGD*, *adult men*, *urban]*

AGYW sometimes described girls who were able to get as much as they could from partners as “savvy”. Both men and older women talked about girls being savvy and entering into sexual relationships with older male partners with a clear intent to obtain specific items. Participants further expressed that, once these girls had achieved their goals, they were no longer interested in the relationship. Most young women admitted using their erotic power in this way, while a few voiced admiration for the strategy:

*They [peers] think she is clever… most girls think that she is savvy… that she knows how to get things from men because whenever she returns home she comes with a gift… That is how she is… I mean within two days*, *she has come back with many things… most people will praise her and say she certainly knows how to extract money*. *[FGD*, *young women*, *rural]*

### Emic perspectives on sexual exploitation inherent in transactional sex

#### Emic conceptualization of sexual exploitation

The Swahili terms that closely align with the English word “exploitation” are *unyanyasaji* [literally meaning “maltreatment”] and *unyonyaji* [literally meaning “to suck”]. Participants used these words interchangeably. The majority of participants described exploitation as referring to sex without the girl’s consent and the use of force or coercion to have sex with her, and where the girl did not receive material or non-material benefits. Other important factors that characterized men exploiting girls in sexual relationships were: young age of the girl versus older age of the man and the man taking advantage of the poor circumstances of the girl.

Despite some recognition of the disadvantaged position of young women, there is nonetheless a sense that exploitation happens in both directions. Most adult women described girls’ experience of exploitation in sexual relationships as self-invited, and the inevitable outcome of their desire for luxuries. One woman reported:

*Exploitation exists because the female child must be exploited* … *she must be exploited because that girl wants to have luxuries at a young age… she wants a good life*. *[IDI*, *adult woman*, *rural]*

#### Intersection between reciprocity norms, gender norms and sexual exploitation

When discussing situations that could be considered exploitative, most girls and adult women could not conceive of a relationship where there was no exchange of gifts or money. In fact, they considered situations where a man had sex with a girl without giving her or promising her any gifts/money as *kuonewa* (“mistreatment”) or *kudharauliwa* (“contempt”). The majority of young women described relationships where men did not provide gifts or money using words such as “disrespectful”, “cruel”, “not good”, “mistreatment” and “exploitative” and denying a girl her “rights”. While describing the social norms of exchange in sexual relationships and what the absence of it meant, adult men and women gave an “emic” description of sexual exploitation as follows:

*According to me*, *sexual exploitation [unyonyaji wa kingono] is when he uses her without any benefit to her in any way* … *It is like you are having sex with a woman and you do not give her any assistance*… *he has not given her*, *her rights*. *[FGD*, *adult women*, *urban]*

Men had a similar view:

*In my view sexual exploitation is when a man has sex with a woman without her wish or he uses her without giving her any benefit… when you are having sex with a woman and you are not giving her any assistance… that is sexual exploitation*. *[IDI*, *adult man*, *urban]*

Since men offering women gifts is considered a gesture of respect and care, not complying with this is considered demeaning to the woman. Drawing on the reciprocity norms, a rural girl described her expectations of receiving from her partner and said that anything not meeting this was exploitative to her:

*I wouldn’t like it if he was just to play with my body without helping me*. *After we meet as partners and he has finished his problem [satisfied his sexual desires]*, *he should also help me with my problem [material needs]*. *It is unfair for him to just have sex with me while he sees me walking bare feet [unable to afford shoes]*, *it is not fair*. *If you care [concerned] for somebody he is supposed to care for you too*. *[IDI*, *out-of-school girl*, *18 years*, *rural]*

Reciprocity norms are further reflected in girls’ expectations about the use of their body. Most participants were in agreement that girls having sex without gifts or money was dehumanizing and equated this to tiring one’s body for nothing. Such “free” sexual encounters were considered stigmatizing to the girl and resulted in labels such as *malaya* (“prostitute”), *bodabod*a (“public transport”) and *jamvi la wageni* (“door mat/visitors mat”) as recounted by adult women in the following:

*If they know that she sleeps with him for free*, *they will gossip about her a lot… “That the daughter of so and so is being seduced for free… she does prostitution* … *And she even wears borrowed clothes”… They will fabricate stories about her… I mean they now label you “bodaboda” meaning that she just gives her body for free*, *without being given anything in return… she just distributes [FGD*, *adult women*, *rural]*

The above quote almost implies that the girl is being “cheap” in giving away her body for free. The use of the label “prostitute” has varied connotations. It can imply an unmarried woman engaging in sex or a young woman having sex for free. Girls who receive nothing in return for sex are doubly stigmatized—both for being sexually active and for being “taken advantage of” or foolish for letting men have something for “free”. Therefore, young men described sex without gifts or money as “foolish prostitution” or rather “prostituting foolishly” (*uhuni wa kijinga*). Young women explained that, in a relationship, a woman had to benefit but also to be able to help her family when in need. A girl who could do so was perceived as a “clever prostitute”. In keeping with this, young women reported:

*That is foolish prostitution [laughter]*, *she doesn’t send money to her family*. *While a clever one fends for her family*, *a foolish one does nothing to help even when her family is going hungry*. *[FGD*, *out-of-school young women*, *rural]*

Further, there is a belief that a woman not requesting anything from her partner is a sign of her being “infected with disease” or not “normal”—implying something is wrong with her. Out-of-school young women reported that men would be scared of such a woman, thinking she has a terrible disease.

*The men feel that the one who asks for money is better because when you are always free of charge they will eventually run away from you… They will say that there is something in her*, *maybe she has diseases… it is not normal to just keep going for free*. *[FGD*, *out-of-school young women*, *rural]*

#### Power differentials and sexual exploitation

Relationships between AGYW and older men are common. Inasmuch as the value of the gifts is important in young women’s sexual decision-making, many participants, especially older women, reported that girls had a choice of whom to have sex with and therefore would only get in relationships with partners they loved/liked and would refuse a gift from men they did not love. However, despite some signs of agency in relationships, the majority of sexual relationships involve hierarchies of power based on gender, socio-economic status and age. Older male partners having money and the young girl needing the money place the latter in a disadvantaged position. Therefore, both parents and young people described relationships involving young women and older men using words such as “It is not right”, “It is exploitative” and “He is ruining the student’s life.” Such encounters are considered exploitative to the girl because the girl is physically and emotionally immature compared with the man and because men usually use money to gain access. Adult women reported:

*You might find that she is still young*, *a man has seen her*, *he likes her so he goes and coerces her by all means*, *he gives her money*, *he buys her many things*, *that is*, *to deceive her because that girl is still young… Her mind is easily enticed… So she enters into sex before the right age*. *[FGD*, *adult women*, *rural]*

Further a secondary school girl talked about the unfairness, power differences and coercion in teacher–student relationship in the following:

*There are those who use their positions… especially the teachers*. *I think it is not good*. *You might get the students who are not ready to have sex with them*, *but the teachers just force them… Now when you come to think about it*, *he is a teacher then he is harsh*, *you just have to have sex with him so that you are not beaten*. *[IDI*, *in-school young woman*, *17 years*, *rural]*

However, the perception of intergenerational sexual relationships as fair/not fair, good/not good is filtered by the perceived “intentions” of the man for the AGYW’s future. There is a social expectation that a “good partner”, in addition to helping the girl meet immediate material needs, will consider her future and show an interest in marrying her—so if a man is with a girl but does not intend to marry her this is assessed negatively. Participants, especially women, talked about adult married men having sex with AGYW in the following:

*He is just playing “on” her because he will not marry her… He is just ruining her life… She might even become pregnant and that becomes a loss* … *it is only women who become pregnant and not men*! *[FGD*, *adult women*, *urban]*

Most participants agreed that it was difficult to ascertain whether a sexual act was consensual or not. Usually, in cases of coercion/rape claims by the girl, the community assesses the circumstances surrounding the encounter. Girls are expected to avoid situations that could lead to forced sex, such as accepting gifts and being with the man in a private place alone. Adult women reported that, if a girl put herself in a situation of temptation, such as by going to a man’s room in private and then refusing to have sex with him, she deserved to be forced. Usually, such girls do not receive sympathy from their community because they are perceived as having invited the abuse.

*She has agreed because if he has forced you*, *I mean he has raped you*, *I mean you should have screamed for the public to hear… But we don’t hear the noises… one should have lamented that “What are you doing*?*”*, *so that means it is completely consensual*. *I mean you have accepted that’s why you are quiet… The one who is forced will make noise and she will get help*. *[FGD*, *adult women*, *urban]*

Men seem to abuse the expectation that they should provide for their partner. Young women described tactics used by older men to get AGYW interested in them, such as exposing their money and/or openly discussing their wealth to entice girls to agree to have sex with them. There was a widespread view that women could not resist sex with a man who appeared to have money. A young woman talked about her experience in the following:

*He convinces you by giving you money*. *First you refuse* … *He again gives you and tells you*, *“Here is money*, *take it”… Once you take*, *you become fond of taking… and there you are caught*. *[IDI*, *out-of-school young woman*, *20 years*, *rural]*

In addition, some school girls talked about men taking advantage of poor girls by coercing them into sex, such as in the following:

*You know our school is far… You find that he is a driver of bodaboda [motorbike] and so he gives her a lift to school… He even fetches her from school*. *He deceives her with little gifts until she agrees to have sex with him*. *[IDI*, *in-school young women*, *17 years*, *urban]*

## Discussion

Our findings offer insights into the social norms and expectations within AGYW’s sexual relationships and how they intersect with exploitation. As observed in other studies [3, 4, 7] and also here, transactional sex is a common aspect of AGYW’s sexual encounters. While community perspectives seem to be in support of women who engage in transactional sex in a particular way, AGYW’s engagement in premarital sex is condoned in Tanzania [8]. Exchange within relationships is governed by male provider norms that dictate that men provide for their female partner. Exploitative aspects of relationships that are linked to transactional sex are: lack of male provision in sexual relationships and coercion tactics used by men in order to have sex with young women.

Exchange within sexual relationships is further perpetuated by reciprocity norms. Based on the principle of reciprocity, which has been a fundamental social structure in many African societies [25, 26], older male partners’ offer of resources gives AGYW the right to receive, and receiving obliges them to offer something in return (agreeing to a sexual encounter). Given the gender inequalities in Tanzania, the expectation for reciprocity seems to affect sexual relationship dynamics perpetuating unequal relationships between older men (with resources) and younger women (with limited resources). These gender inequalities further point to imbalanced reciprocity. Modernity and globalization have also generated more pressure on AGYW to consume certain goods and look a certain way, fuelling their desire to obtain those goods and the associated status, but they are still constrained by their lack of access to resources. In this situation, AGYW agentically resort to having sex with older partners as a way of achieving these goals. As noted by Luke et al. [2], AGYW who depend on resources from male partners are likely to be weak in the negotiation of sex, including condom use. This is exacerbated when the male partner is older than the girl because, as the amount of money exchanged increases, the likelihood of consistent condom use reduces [2].

Similar to what was found in this study, other studies have observed compliance by women and complicity with gender norms [27]. Our findings indicate that AGYW’s compliance with social norms to engage in transactional sex is linked to their desire or attempt to be perceived favorably by the community (especially peers). This suggests that compliance with transactional sex may be linked to a certain female gender identity promoted by the community, that of girls who engage in the practice for something (not for free). This in turn perpetuates men’s use of their economic advantage over AGYW with least resources.

While several studies have found that sexual exploitation continues to be a common problem for many girls globally [21, 28], we found that community perspectives on transactional sex and exploitation varied considerably and were sometimes contradictory. The circumstances surrounding a relationship are critical in determining if a particular incident is exploitative, fair or not fair, right or wrong. Whether an incident of transactional sex is considered exploitative or not depends on factors such as the difference in age between the girl and the partner, a man’s intention of carrying out the exchange, the motive and long-term plans of the partner for the girl (e.g. marriage) and the perceived benefits of the exchange and the circumstances leading to the sexual encounter. Under the United Nations Convention on the Rights of the Child, anyone under the age of 18 is still considered a child [29]. While some authors may classify transactional sex as exploitative [21, 29], some report that girls specify psychosocial benefits such as feelings of love and increased self-esteem and self-confidence among peers, as well as elevated social status among peers [9, 30, 31] as influenced by globalization norms.

It is apparent in our findings that most girls and their communities may not necessarily consider girls who engage in transactional sex as exploited. In fact, the few young women who engage in sexual encounters that do not involve receiving something are stigmatized and referred to using labels such as *bodaboda* (“public transport”) and *malaya* (prostitute). So, even though the community recognizes a link between reciprocity and exploitation of women, it does not seem to react to the “unfairness” of not getting anything in return for sex but judges women’s characters—demeaning them as the “worst type of prostitute” (compared to a “better” one), “diseased” and “doormats”. This is evidence of the social sanctions directed towards women who do not conform to a complacent configuration of femininity that sees as its duty to provide sex in exchange for provision from men. It also highlights the intersection between reciprocity and exploitation, as it seems that the community interpretation of exploitation is heavily influenced by social norms that dictate gendered ways to enact the exchange of sex for material goods.

Stigmatizing labels like these point to community expectations for women to transact in specific ways. Such labels also encourage young women to engage in transactional sex regardless of the consequences of the practice. While these labels refer to the literal meaning of sex work, they are used paradoxically to refer to women who have sex without receiving any gifts and/or money from their partners. These labels may complicate efforts endeavoring to distinguish transactional sex from sex work [7]. Nonetheless, they offer useful insights for understanding social norms perpetuating transactional sex in Tanzania. This finding clearly points to the need to understand the social norms driving risky practices and what the absence of this practice might mean for the communities involved.

Inasmuch as communities have their own understanding of what constitutes sexual exploitation in AGYW’s relationships or sexual encounters, they do little to stop its occurrence. A girl’s behavior, in particular respectful conduct, is important in determining the level of sympathy she receives from the community in the event that she experiences unfair treatment from males. Instances where there is no sympathy with the occurrence of coercion include when a girl benefits from the man but always declines sex.

Evidence shows that girls who are forced to have sex against their will or because they feel they need to have sex to meet basic needs are at an increased risk of intimate partner violence and other sexual and reproductive health outcomes, including HIV [4, 13]. Our findings indicate that structural factors such as living in poverty push AGYW into vulnerability and manipulation by sexual partners through transactional sex. Other studies have also noted a link between transactional sex and coercion [32] and condom use [2, 33]. There is a need to address the pressure young people face from adult men to have sex in exchange for goods, money, grades at school or employment and the limits these gendered power dynamics place on their ability to negotiate sex.

Social power is very important for the sexual decision-making of both men and women in the study setting. This social power could be a result of social status (e.g. teacher) or of money (e.g. businessman). Hence, girls agree to have sex with men who have either economic or social power. Our results show that wealthier men (teachers, businessmen) use their wealth and resources to convince girls to have sex in exchange for money and gifts, including favors. These exchanges are problematic for girls who are poor and striving to meet basic needs. Studies have noted similar findings in other settings in sub-Saharan Africa where girls rely on men for basic needs and where men take advantage of girls because of the latter’s economic needs [7, 21].

The perception that girls who take advantage of men are savvy suggests that many of the AGYW are not passive victims in their relationships, but rather are making strategic choices within the structural constraints of their realities. Some authors have importantly called for the need to recognize the agentic nature of using one’s sexuality as a means to achieve objectives [5, 9]. However, it must be noted that not all agency, the latter understood as the act of deciding to take a course of action, is positive or transformative [34]. Some agentic behavior may result in mixed outcomes. In this case in particular, girls may obtain the desired goods, which are otherwise obtainable given their limited access to resources in these societies, but at the same time the pervasiveness of transactional sex may perpetuate a *status quo* of gender power imbalances. Finding the right balance between acknowledging this agency and contextualizing it within the structural constraints imposed on it is key if we want to go beyond principalist debates and really think about the best way of designing programs that help girls and young women live healthier lives and achieve their goals.

As with all qualitative studies, our relatively small non-random sample makes it difficult to generalize the results. We acknowledge that the sample size may limit our ability to confidently compare themes across the various sub-groups. Moreover, inasmuch as snowball sampling has strengths, such as ensuring homogenous groups (key for FGDs), its use may have led us to a specific social network of individuals, which may have biased some of the results. However, the use of maximum variation sampling procedure meant we were able to capture viewpoints from different types of participants (adult women and men and young men and women, in and out of school, in rural and urban settings). Although there were no clear differences in findings in urban and rural settings, by focusing on a limited number of communities we were able to gain deeper insights into social norms influencing transactional sex and how they influence people’s perceptions of sexual exploitation of girls. We therefore gathered context-specific information that will be useful in developing country settings with similar characteristics to rural and urban Tanzania.

## Conclusion

Interventions aimed at reducing AGYW’s exploitation through transactional sex need to be cognizant of the variations in communities’ understanding of: what constitutes AGYW’s sexual exploitation; and the social and gender norms influencing the practice of transactional sex and people’s perception of the practice. From our results, it becomes clear that what is considered exploitative differs according to the circumstances surrounding the relationship and the character of the AGYW. Therefore, exploitation cannot be defined in a vacuum; it needs to be interpreted against a backdrop of norms and expectations that shape the experience of people involved as exploited or not, and how they experience this exploitation.

From our data it also becomes apparent that, even within exploitative situations in sexual relationships, there are benefits for both sides. If we are unable to recognize this, interventions aimed at improving AGYW’s health and social well-being will fail, as they will not resonate with the subjective experience of their intended beneficiaries. Communities need to be involved in a critical reflection exercise of aspects of transactional sex that could be exploitative and encouraged to help protect AGYW who report being exploited, regardless of the circumstances of their relationship or their character.

Furthermore, interventions need to be sensitive to the structural dynamics that underlie sexual exploitation (e.g. through transactional sex, girls’ economic vulnerability). Given the pressure girls face from peers, parents and the men who seek sex with them, they alone are unlikely to have the power to avoid exploitative situations in their lives without a change in the support around them. Hence, interventions should adopt mobilization strategies (involving whole communities and families) aimed at bolstering, shifting or introducing positive alternative norms at the community level [35] that could help counter the harmful gendered norms that shape the involvement of AGYW and men in transactional sex. Finally, interventions need to address the social expectations related to certain ways of obtaining material goods to keep up with modern lifestyles.
